# Polyphenols and Polysaccharides from *Morus alba* L. Fruit Attenuate High-Fat Diet-Induced Metabolic Syndrome Modifying the Gut Microbiota and Metabolite Profile

**DOI:** 10.3390/foods11121818

**Published:** 2022-06-20

**Authors:** Meixia Wan, Qing Li, Qianya Lei, Dan Zhou, Shu Wang

**Affiliations:** 1 West China School of Pharmacy, Sichuan University, Chengdu 610041, China; wanmeixia2010@126.com (M.W.); 18381476874@163.com (Q.L.); lqy2205@126.com (Q.L.); wendyzz12@163.com (D.Z.); 2 Qibo College of Medicine, Longdong University, Qingyang 745000, China

**Keywords:** *Morus alba* L. fruit, polyphenols, polysaccharides, metabolic syndrome, gut microbiota, untargeted fecal metabolomics

## Abstract

*Morus alba* L. fruit, a medicinal and edible fruit in East Asia, showed potential health-promoting effects against metabolic syndrome (MetS). However, both the protective effects and mechanisms of different fractions extracted from *Morus alba* L. fruit against MetS remain unclear. Additionally, the gut microbiota and its metabolites are regarded as key factors in the development of MetS. This study aimed to investigate the potential role of polyphenols and polysaccharides derived from *Morus alba* L. fruit against MetS in high-fat diet (HFD)-fed mice, individually and in combination, focusing on remodeling effects on gut microbiota and metabolite profiles. In the study, polyphenols and polysaccharides derived from *Morus alba* L. fruit improved the traditional pharmacodynamic parameters of MetS, including reductions in body weight (BW) and fat accumulation, improvement in insulin resistance, regulation of dyslipidemia, prevention of pathological changes in liver, kidney and proximal colon tissue, and suppressive actions against oxidative stress. In particular, the group treated with polyphenols and polysaccharides in combination showed better efficacy. The relative abundance of beneficial bacterial genera *Muribaculum* and *Lachnospiraceae_NK4A136_group* were increased to various degrees, while opportunistic pathogens such as *Prevotella_2*, *Bacteroides*, *Faecalibacterium* and *Fusobacterium* were markedly decreased after treatments. Moreover, fecal metabolite profiles revealed 23 differential metabolites related to treatments with polyphenols and polysaccharides derived from *Morus alba* L. fruit, individually and in combination. Altogether, these results demonstrated that polyphenols and polysaccharides derived from *Morus alba* L. fruit attenuated MetS in HFD-fed mice, and improved the gut microbiota composition and fecal metabolite profiles.

## 1. Introduction

MetS is a serious issue in public health management [1], as approximately 20–30% of adults suffer from MetS worldwide [2,3]. Possessing a multifactorial origin that is not exactly clarified, it is a so-called “deadly quartet”, composed of obesity, glucose intolerance, hypertriglyceridemia, and hypertension [4]. Previous studies have demonstrated that MetS is related with various clinical disorders, including oxidative stress, pro-inflammatory states, hepatosclerosis and nonalcoholic fatty liver disease (NAFLD) [5]. Additionally, there is an association between the number of metabolic syndrome components and increased risk of cardiac death [6]. As a consequence of its complex pathogenesis, there are no effective therapeutic drugs available [7]. Therefore, it is a high priority to investigate new preventions or treatment drugs.

Gut microbiota, a complicated ecosystem consisting of 10–100 trillion microorganisms from more than 1000 species, serves an essential role in human homeostasis [8]. Dysbacteriosis is related to multiple diseases, and the imbalance may be a consequence of symbiotic flora deficiencies, diversity reduction of normal symbiotic flora, and proliferation of pathogenic bacteria [9]. Additionally, evidence from microbiome studies indicates that the mechanisms leading to risk factors for MetS may originate in the gut, mainly involving the intestinal microbiota and its metabolites [10].

According to *Bencao Gangmu* (A.D.1578–1593) and *Donguibogam* (A.D. 1613), *Morus alba* L. fruit has been a traditional plant with many applications including the ability to alleviate hyperglycemia and hypertension, enhance liver and kidney function, remedy fever, strengthen the joints and moisten dryness and other disorders for thousands of years in East Asia [11,12]. In the Chinese pharmacopoeia version of 2020, *Morus alba* L. fruit is used for the treatment of liver and kidney deficiency, dizziness and tinnitus, palpitation and insomnia, premature graying hair, diabetes, constipation and intestinal dryness [13]. *Morus alba* L. fruit contains many active ingredients, such as polyphenols and polysaccharides, the former including flavonoids and anthocyanins [14], which function as natural antioxidants in the human body. Polyphenols are abundant micronutrients in a well-balanced diet. Dark-colored fruits and vegetables contain substantial levels of anthocyanins. The major anthocyanin compound in *Morus alba* L. fruit is cyanidin-3-*O*-glucoside [15]. Anthocyanin is a naturally occurring pigment that scavenges free radicals in vivo, conveying health functions that are anti-inflammatory, weight-reducing, and anti-tumor, as well as preventing metabolic disorders [16]. Natural polysaccharides, one of the most abundant dietary components, have versatile nutraceutical effects, such as immunomodulatory, antioxidant, anti-inflammatory, anti-tumor, and hypoglycemic effects [17]. Of note, dietary polyphenols and polysaccharides, which are potent modulators of gut microbiota composition, have been shown to benefit patients with metabolic syndrome [5,17].

In previous studies, it was shown that *Morus alba* L. fruit powder or crude extracts could prevent some components of MetS, such as dyslipidemia, hyperglycemia, obesity, NAFLD and tumors [18,19]. Previous studies on the mechanisms of action of *Morus alba* L. fruit extracts have focused on the following: upregulating the antioxidant activities and p-CREB/BDNF pathway [20], activation of PI3K/Akt and AMPK signaling pathways [21] and lessening oxidative stress and inflammation [22]. Epidemiological surveys and nutritional interventions with anthocyanins and polysaccharides have demonstrated beneficial effects in patients with metabolic syndrome-related chronic diseases [23,24]. However, the protective effect of different fractions extracted from *Morus alba* L. fruit against MetS, as well as the mechanisms of *Morus alba* L. fruit in preventing MetS from the perspective of gut microbiota and metabolomics, have not been fully illustrated. In this study, we aimed to clarify the potential preventive mechanisms of polyphenols and polysaccharides derived from *Morus alba* L. fruit against MetS in HFD-fed C57BL/6J mice. The preventive effects of the polyphenols, polysaccharides and their combined intervention were evaluated by serum biochemical indexes and pathological changes in liver, kidney and proximal colon tissue. Integrated 16S rDNA sequencing and untargeted fecal metabolomics were used to elucidate the variations of intestinal flora and their metabolic profiles.

## 2. Materials and Methods

### 2.1. Extraction, Isolation, and Purification of Morus alba L. Fruit Polyphenols and Polysaccharides

Dried *Morus alba L.* fruit was provided by Sichuan Heijinshen Sunshine Agriculture Co., Ltd. (Yanbian County, Panzhihua City, Sichuan Province, China) in 2020 and identified by Dr. Wang (professor in Department of Pharmacognosy, West China School of Pharmacy, Sichuan University). A voucher specimen (No. 20200628-2) has been deposited in the Herbarium of West China School of Pharmacy, Sichuan University.

*Morus alba* L. fruit powder was extracted with 85% ethanol/water solution under percolate conditions and stored in a dark area for 24 h. The extract was filtered and evaporated (45 °C) to obtain an aqueous residue. Then, the aqueous residue was purified by AB-8 macroporous adsorption resin (Tianjin, China), eluted with 90% ethanol, and the brunette eluent was collected. The polyphenol-rich fraction (*Morus alba* L. fruit polyphenols, MFP) was obtained by evaporating the ethanol at 45 °C and drying in a freeze-dryer.

*Morus alba* L. fruit powder was extracted with purified water at 1:10 (*w*/*v*), and the extraction temperature was controlled at 90 °C for 2 h. The Savage method (n-butanol: chloroform = 1:4 *v*/*v*) was applied to separate protein. The extract was evaporated at 60 °C, and the concentrated solution was supplemented with 5 volumes of 95% ethanol. After 24 h at room temperature, the precipitate was collected and freeze-dried, and finally the polysaccharide fraction (*Morus alba* L. fruit polysaccharides, MFS) was obtained.

### 2.2. Compositional Identification of MFP and MFS

HPLC analysis was performed on a Shimadzu SPD-10A system (Shimadzu Corp., Kyoto, Japan) to analyze the composition of MFP. HPLC-grade acetonitrile and phosphoric acid were purchased from Merck Co., Ltd. (Darmstadt, Germany) and Tianjin Kemiou Chemical Reagent Co., Ltd. (Tianjin, China), respectively. Ultrapure water was obtained from an Ultrapure water machine (UPR-II-10T, Chengdu, Sichuan). Cyanidin-3-*O*-glucoside chloride (batch number: PS011320) was purchased from Push-Herbchem Co., Ltd. (Chengdu, Sichuan). Rutin (batch number: 100080-201811), gallic acid (batch number: 110831-201605) and D-glucose anhydrous (batch number: 110833-202109) were purchased from the National Institutes for Food and Drug Control (Beijing, China). The gradient elution mobile phases consisted of acetonitrile (A) and 0.5% aqueous phosphoric acid (B) on a Sepax MAH-C18 column (250 mm × 4.6 mm; 5 μm) at a flow rate of 1.0 mL/min. The gradient profile was as follows: 0–10 min, 10–12% (A); 10–30 min, 12–15% (A); 30–40 min, 15–30% (A); 40–45 min, 30–10% (A). The column temperature was controlled at 30 °C, and the wavelength of the UV detector was 520 nm. The injection volume was 10 μL. Total polyphenols and anthocyanins for MFP and total polysaccharides for MFS were determined using an ultraviolet (UV) spectrophotometer (Alpha-1860, Shanghai, China) according to the methods previously described [25].

### 2.3. Animal Treatment

The animal experiment was approved by the Ethics Committee of Sichuan University, Chengdu, China (protocol number: SYXK (Chuan) 2018-113), and all procedures followed European Community guidelines (86/609/EEC) for the Care and Use of Laboratory Animals. Seventy–two male C57BL/6J mice (18 to 20 g) were obtained from Chengdu Dossy Experimental Animals Co., Ltd. (Chengdu, China) (SCXK (Chuan) 2020–030). Simvastatin tablets were purchased from Shandong Lukang Group Saite Co., Ltd. (20 mg/tablet, Tai’an, China). Food-grade fructose was purchased from Zhejiang Yino Biotechnology Co., Ltd. (Quzhou, China). Mice were maintained under SPF conditions at room temperature 22 ± 2 °C and relative humidity 50 ± 5%. Before the experiment, mice were housed in standard conditions with free access to food and water for 8 days. All mice were randomly assigned into six groups (*n* = 12 per group, three mice per cage), including normal chow diet (NCD) group (same volume of purified water), high-fat diet (HFD) group (same volume of purified water), PC (positive control) group (simvastatin suspended in 0.5% CMC-Na, 2.6 mg∙kg^−1^, BW) [26], MFP group (300 mg∙kg^−1^, BW), MFS group (600 mg∙kg^−1^, BW), and MFPS group (300 mg∙kg^−1^ MFP+ 600 mg∙kg^−1^ MFS, BW). Mice were administered intragastrically once a day (9:00–11:00 a.m.) for 14 weeks. During the experimental period, mice in the HFD, PC, MFP, MFS and MFPS groups were fed with high-fat diet (45% caloric fat, purchased from BiotechHD Co., Ltd. (Beijing, China)) and 4.2% fructose drinking water, while the mice in the NCD group were fed with normal chow diet (15% caloric fat, purchased from the Chengdu Dossy Experimental Animals Co., Ltd., Chengdu, Sichuan, China) and purified water. Body weight was monitored weekly. Prior to sacrifice, all mice were fasted for 12 h from 9:00 a.m. to 9:00 p.m., while ensuring they had access to adequate water.

### 2.4. Oral Glucose Tolerance Test (OGTT)

OGTT was performed at the end of the 14th week of intervention. Mice were fasted overnight for 12 h and then given glucose intragastrically 2 g∙kg^−1^, BW). Blood was collected from the caudal vein, and levels of glucose were analyzed with ACCU-CHEK^®^ Performa glucometer (Roche Diabetes Care GmbH, Indianapolis, IN, USA) before (0 min) and after (30, 60, 90 and 120 min) gastric glucose gavage.

### 2.5. Sample Collection

Fecal samples were collected in duplicate by stimulating the anus of mice on a sterile clean bench. After anesthetizing under ether, blood was collected through eye orbit, and centrifuged at 3000× *g* for 10 min at 4 °C to obtain the serum samples. The liver, kidney, epididymal adipose and colon were quickly dissected. Feces, serum and tissues were immediately frozen in liquid nitrogen and stored at −80 °C until further analysis.

### 2.6. Biochemistry Assays and Histological Examination

The activities of aspartate aminotransferase (AST) and alanine amino transferase (ALT), as well as the levels of total cholesterol (TC), triglyceride (TG), low density lipoprotein-cholesterol (LDL-C), high density lipoprotein-cholesterol (HDL-C), blood urea nitrogen (SCr), serum uric acid (SUA) and blood urea nitrogen (BUN) in the serum were measured by an automated biochemical analyzer (Olympus AU400, Tokyo, Japan). Serum insulin levels were detected by mouse insulin ELISA kit (Shanghai Jianglai Industrial Limited By Share Co., Ltd., Shanghai, China). The homeostasis model assessment of insulin resistance (HOMA-IR) was evaluated according to the formula fasting insulin (mIU/L) × fasting blood glucose (mmol/L)/22.5. The concentrations of interleukin-1β (IL-1β), interleukin-6 (IL-6) and tumor necrosis factor-α (TNF-α) in serum were measured by ELISA kits (Thermo Fisher Scientific, Wilmington, DE, USA). Livers were homogenized with 0.9% saline at a ratio of 1:10 (*w*/*v*) and centrifuged (3000× *g*, 10 min). The levels of malondialdehyde (MDA), superoxide dismutase (SOD) and glutathione peroxidase (GSH-Px) in liver homogenates were determined by available kits (Nanjing Jiancheng Institute of Biological Engineering, Nanjing, China).

### 2.7. Histological Analysis

Liver, left kidney, proximal colon and epididymal adipose tissue were fixed in 4% paraformaldehyde (Servicebio, Wuhan, China) for 48 h, embedded in paraffin, sectioned at 5 μm and stained with hematoxylin and eosin (H&E). The left lobe of liver sections was stained with oil red O in order to analyze the accumulation of fat in liver. The fixed left liver lobe was dehydrated with 30% sucrose solution until the tissue sank, then embedded with optimal cutting temperature compound (OCT) and stored at −80 °C for 12 h before being sectioned. Finally, the sections were stained with oil red O. The stained sections were investigated with an optical microscope (Olympus BX53, Tokyo, Japan).

### 2.8. Gut Microbiota Analysis by 16S rDNA Gene Sequencing

Frozen mouse feces in each cage (three mice) were mixed in equal concentrations as a sample. The genomic DNA was extracted from feces samples by the MagPure Soil DNA LQ Kit (Magen, Guangzhou, China). The concentration and purity of DNA were assessed by NanoDrop™ 2000 UV-Vis spectrophotometer (Thermo Fisher Scientific, Wilmington, DE, USA). The V3-V4 regions of 16S rDNA were amplified by PCR (Bio-Rad, Hercules, CA, USA) by using primers with barcodes (338F: ACTCCTACGGGAGGCAGCAG/806R: GGACTACHVGGGTWTCTAAT) and Tks Gflex™ DNA Polymerase (R060B, Takara, Japan). Amplicons were visualized by gel electrophoresis and clarified with AMPURE^®^ XP beads (Agencourt, Pasadena, CA, USA). A Qubit™ dsDNA HS Assay Kit (Q32854, Life Technologies, Eugene, OR, USA) was used to quantify the amplicons. An equivalent quantity of clarified amplicons was pooled for the following sequencing. The library was structured in accordance with standard operational protocols of the Illumina MiSeq^®^ Platform (Illumina, San Diego, CA, USA). The sequencing was performed by the MiSeqPE300 Illumina platform. Original data were preprocessed, and ambiguous bases (N) were trimmed (Trimmomatic, version 0.35). Then, the spliced sequences were assembled by FLASH software (version 1.2.11). Clean tags were removed and clustered to produce OTUs at a level of 97% similarity (vsearch, version 2.4.2). High-quality sequences (validated tags) were extracted by QIIME package (version 1.8.0) and annotated against the Silva database (version 132).

### 2.9. Untargeted Fecal Metabolomics Analysis

Frozen mouse feces in each cage (three mice) were mixed in equal concentrations as a sample. Fecal metabolites were analyzed by Thermo Scientific Q Exactive Plus with Dionex UltiMate 3000 UHPLC (Thermo Fisher Scientific, Wilmington, DE, USA) equipped with an H-ESI source. The chromatographic separation was conducted on an ACQUITY UPLC HSS T3 column (1.8 μm, 2.1 × 100 mm, Waters, Milford, MA, USA), and the column temperature was set at 45 °C. The mobile phase was composed of 0.1% formic acid (A) and acetonitrile (B). The gradient elution was carried out as the following procedure: 0–2 min, 5% B; 2–4 min, 5% B; 4–8 min, 30% B; 8–10 min, 50% B; 10–14 min, 80% B; 14–15 min, 100% B; 15 min, 100% B; 15.1–16 min, 5% B. The injection volume was 2 μL, and the flow rate of the mobile phase was 0.35 mL/min.

The resolutions of full mass spectrometry scan and HCD MS/MS scan were set at 70,000 and 17,500, respectively. The mass spectrum was recorded in the range *m*/*z* 100–1000, and collision energy was generated at 10, 20 and 40 eV. The mass spectrometric settings were manipulated with the following parameters: spray voltage, 3800 V (+)/3000 V (−); sheath gas flow rate, 35 arbitrary units (arb); auxiliary gas flow rate, 8 arb; capillary temperature, 320 °C; aux gas heater temperature, 350 °C; s-lens RF level, 50. The QCs were analyzed at regular intervals (every five fecal samples) to assess stability during the analytical procedure.

### 2.10. Statistical Analyses

The experimental data were statistically analyzed by GraphPad Prism 7.0, SPSS 20.0 and R 4.1.0, and presented as means ± standard deviation. Statistical significance was evaluated by one-way analysis of variance (one-way ANOVA). The means of the two groups were tested through an independent Student’s *t*-test. The raw spectra were processed by metabolomics processing software (Progenesis QI V2.3, Nonlinear Dynamics, Newcastle, UK). For all tests, *p* < 0.05 was considered statistically significant.

## 3. Results

### 3.1. Identification Analysis of MFP and MFS

**Figure 1:**
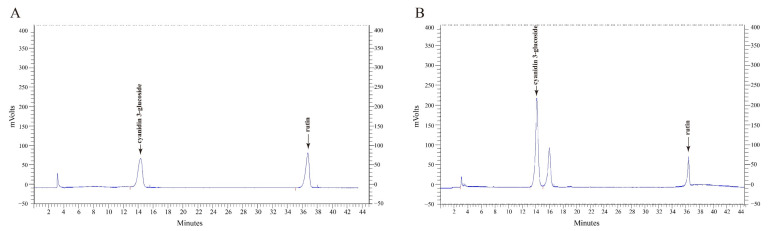
Identification analysis of MFP. (**A**) HPLC analysis of cyanidin-3-*O*-glucoside and rutin. (**B**) HPLC analysis of MFP.

Total polyphenols and anthocyanins of MFP reached 772.3 mg/g and 361.2 mg/g, respectively. Total polysaccharides of MFS reached 854.1 mg/g. The HPLC chromatograms of MFP are shown in Figure 1A,B. The concentrations of cyanidin-3-*O*-glucoside and rutin in MFP reached 172.7 mg/g and 32.5 mg/g, respectively.

### 3.2. Alleviation of Obesity and Fat Accumulation in HFD-Fed C57BL/6J Mice after MFP, MFS and MFPS Treatments

**Figure 2:**
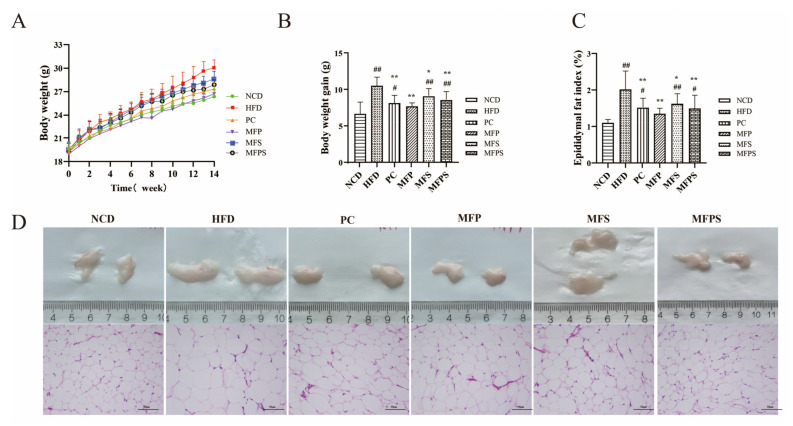
The improvement of obesity and fat accumulation in HFD-fed mice after MFP, MFS and MFPS treatments. (**A**) Changes in body weight. (**B**) Body weight gain. (**C**) Epididymal fat index. (**D**) Epididymal adipose tissue and H&E-stained photomicrograph of epididymal adipose tissue (original magnification × 200). Data are expressed as the mean ± standard deviation (*n* = 12). *^#^ p* < 0.05, *^##^ p* < 0.01 vs. NCD group; * p < 0.05, ** p < 0.01 vs. HFD group.

During the 14-week feeding period, the body weight of mice in all groups exhibited an ascending trend (Figure 2A). Meanwhile, compared with the HFD group, mice in the PC, MFP, MFS and MFPS groups experienced considerable reductions in body weight gain and epididymal fat index (Figure 2B,C) (*p* < 0.05), while there were no meaningful differences between MFP and NCD groups (*p* > 0.05). The H&E staining demonstrated that epididymal adipocyte volume was enlarged in the HFD group compared with the NCD group. Additionally, the epididymal adipocyte volume was reduced in the PC, MFP, MFS and MFPS groups compared with the HFD group (Figure 2D). It was indicated that MFP, MFS and MFPS significantly inhibited HFD-induced body weight gain and fat accumulation, and the effects of MFP and MFPS were similar to simvastatin.

### 3.3. Prevention of HFD-Induced Hepatic Fat Deposition and Oxidative Stress after MFP, MFS and MFPS Treatments

**Figure 3:**
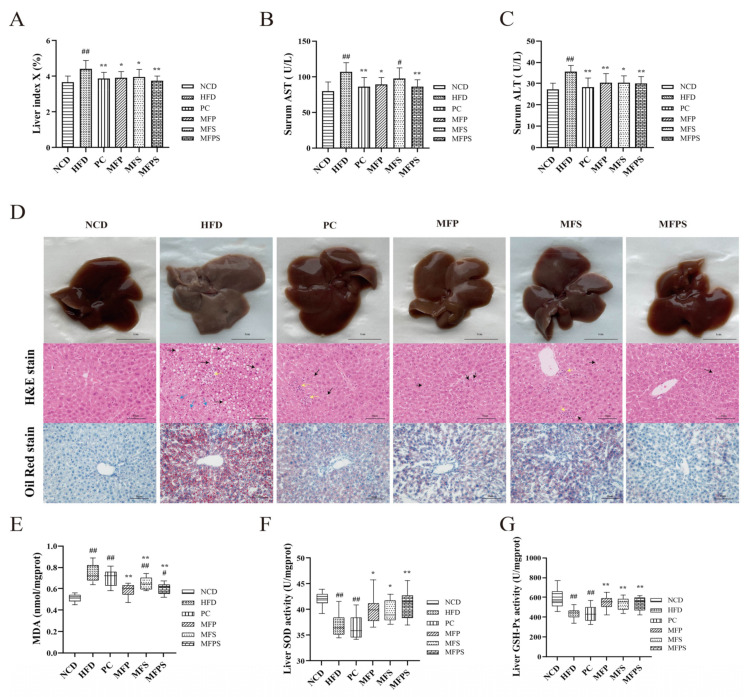
Prevention of HFD-induced hepatic fat deposition and oxidative stress after MFP, MFS and MFPS treatments (**A**) Liver index. (**B**,**C**) Liver function-related transaminase (AST and ALT in serum). (**D**) Macroscopic appearance of liver, H&E-stained photomicrograph of liver tissue and oil red O-stained photomicrograph of liver tissue (original magnification × 200). (**E**) Malondialdehyde (MDA). (**F**) Superoxide dismutase (SOD). (**G**) Glutathione peroxidase (GSH-Px). Black arrows indicate lipid droplets, blue arrows indicate steatosis with edema, and yellow arrows indicate inflammatory cell infiltration. Data are expressed as the mean ± standard deviation (*n* = 12). *^#^ p* < 0.05, *^##^ p* < 0.01 vs. NCD group; * p < 0.05, ** p < 0.01 vs. HFD group.

The activities of AST and ALT in serum are considered to be conventional biochemical markers to determine the degree of liver injury [27]. After administration of simvastatin, MFP, MFS and MFPS, the liver indexes and activities of AST and ALT were reduced (Figure 3A–C) (*p* < 0.01, *p* < 0.05, *p* < 0.05 and *p* < 0.01 for liver index; *p* < 0.01, *p* < 0.05, *p* > 0.05 and *p* < 0.01 for AST; *p* < 0.01, *p* < 0.01, *p* < 0.05 and *p* < 0.01 for ALT), and macroscopic appearances of livers were also improved (Figure 3D). Hematoxylin and eosin (H&E) staining and oil red O staining showed that MFP, MFS and MFPS treatments ameliorated the lipid vacuoles and lipid droplets in liver induced by HFD; in particular, the livers of MFPS-treated mice recovered to near-normal morphology (Figure 3D). It was indicated that MFP, MFS and MFPS alleviated liver fat deposition.

The MDA level in liver tissues, a hallmark of oxidative modification to membrane lipids, was substantially increased in the HFD group and was reduced substantially by MFP, MFS and MFPS treatments (*p* < 0.01, Figure 3E). Compared with the HFD group, SOD activity was evidently enhanced in the MFP, MFS and MFPS groups (*p* < 0.05, *p* < 0.05 and *p* < 0.01), and GSH-Px activity was obviously enhanced (Figure 3F,G). Current evidence suggests that MFP, MFS and MFPS suppress HFD-induced oxidative stress in vivo.

### 3.4. Improvement in Glucose Metabolism Disorder after MFP, MFS and MFPS Treatments

**Figure 4:**
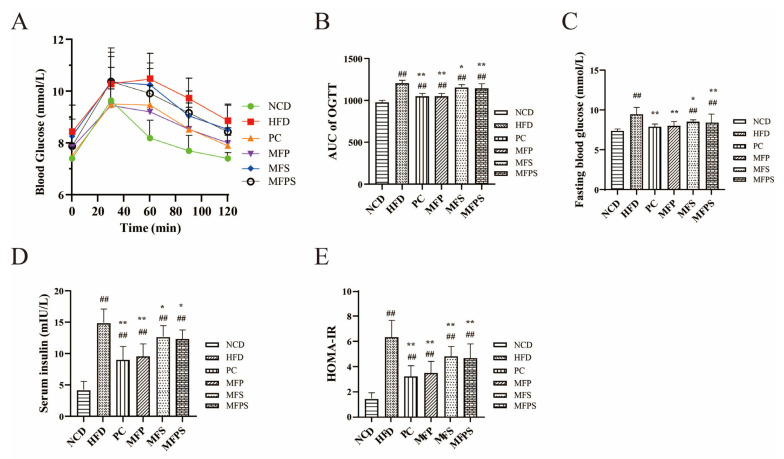
The improvement of glucose tolerance and insulin resistance (IR) after MFP, MFS and MFPS treatments. (**A**) Oral glucose tolerance test (OGTT). (**B**) Area under the curve (AUC) of OGTT. (**C**) Fasting blood glucose. (**D**) Fasting serum insulin. (**E**) Homeostatic model assessment-insulin resistance (HOMA-IR) index. Data are expressed as the mean ± standard deviation (*n* = 12). *^##^ p* < 0.01 vs. NCD group; * p < 0.05, ** p < 0.01 vs. HFD group.

To investigate the effectiveness of MFP, MFS and MFPS on glucose tolerance, OGTT was carried out at the end of the animal experiment. As compared with the HFD group, the glucose tolerance was better in the MFP, MFS and MFPS groups (Figure 4A). Fasting blood glucose, area under the curve (AUC) of OGTT and fasting serum insulin were visibly reduced in the PC, MFP, MFS and MFPS groups (*p* < 0.05) (Figure 4B–D). HOMA-IR parameters were significantly lower after simvastatin, MFP, MFS and MFPS treatments compared with the HFD group (*p* < 0.01 all) (Figure 4E). The above results show that glucose metabolism was disturbed by HFD feeding, and simvastatin, MFP, MFS, MFPS treatments modified the impaired glucose tolerance and HOMA-IR; in particular, the simvastatin and MFP interventions were more effective.

### 3.5. Prevention of Dyslipidemia after MFP, MFS and MFPS Treatments in HFD-Fed Mice

**Figure 5:**
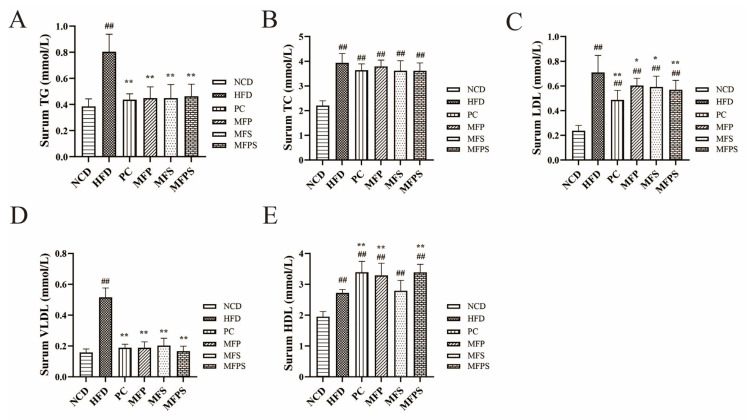
The improvement of hyperlipemia in HFD-fed mice after MFP, MFS and MFPS treatments. (**A**) Levels of total triglyceride (TG). (**B**) Total cholesterol (TC). (**C**) Low-density lipoprotein cholesterol (LDL-C). (**D**) Very low-density lipoprotein cholesterol (VLDL-C). (**E**) High-density lipoprotein cholesterol (HDL-C). Data are expressed as the mean ± standard deviation (*n* = 12). *^##^ p* < 0.01 vs. NCD group; * p < 0.05, ** p < 0.01 vs. HFD group.

It is well established that disturbance in the serum lipid profile is an indication of MetS [28]. Compared with the HFD group, levels of TG, LDL-C, VLDL-C and HDL-C showed significant alterations in the PC, MFP, MFS and MFPS groups (*p* < 0.05), while the HDL-C level was not significantly elevated in the MFS group (*p* > 0.05) (Figure 5A–E). The above results indicated that MFP, MFS and MFPS improved HFD-induced dyslipidemia, but had no effect on TC.

### 3.6. Alleviation of Renal Injury Phenotypes in HFD-Fed Mice after MFP, MFS and MFPS Treatments

**Figure 6:**
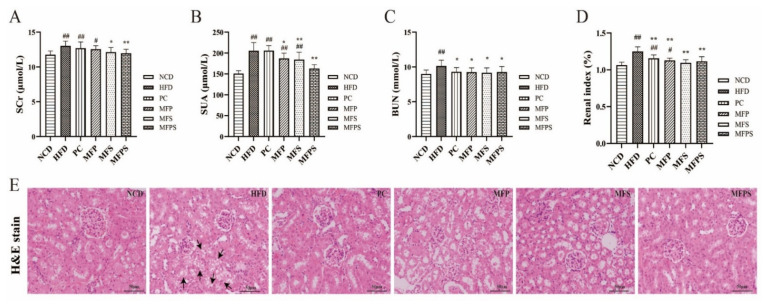
The improvement of renal injury phenotypes in HFD-fed mice after MFP, MFS and MFPS treatments. (**A**) Serum creatinine (Scr). (**B**) Serum uric acid (SUA). (**C**) Blood urea nitrogen (BUN). (**D**) The renal index (%). (**E**) H&E-stained photomicrograph of kidney tissue (original magnification × 200). Black arrows indicate diffuse swelling. Data are expressed as the mean ± standard deviation (*n* = 12). *^#^ p* < 0.05, *^##^ p* < 0.01 vs. NCD group; * p < 0.05, ** p < 0.01 vs. HFD group.

An association between MetS and early renal dysfunction has been realized [3]. Components of MetS and impaired glucose tolerance are risk factors for the progression of kidney disease [29]. After MFP, MFS and MFPS treatments, SCr, SUA and BUN were reduced substantially (*p* > 0.05, *p* < 0.05 and *p* < 0.01 for SCr; *p* < 0.05, *p* < 0.01 and *p* < 0.01 for SUA; *p* < 0.05, *p* < 0.05 and *p* < 0.05 for BUN), while only the level of BUN was lowered in the PC group (*p* < 0.05), in comparison with the HFD group (Figure 6A–C).

As shown in Figure 6D, the treatments with simvastatin, MFP, MFS and MFPS decreased the renal index in comparison with that of the HFD group (*p* < 0.01 all). By H&E staining, partial glomerular atrophy and vacuolar degeneration of renal tubular epithelial cells were observed in the HFD group, indicating potential lipid lesions in kidney (Figure 6E). However, PC, MFP, MFS and MFPS treatments ameliorated HFD-induced vacuolar degeneration in kidney. Furthermore, MFS and MFPS-treated mice exhibited near-normal kidney morphology. Here, it was revealed that MFP and especially MFS and MFPS suppressed the HFD-induced renal injury phenotypes.

### 3.7. Effects of MFP, MFS and MFPS on Inflammatory Mediators and Colonic Lesion Phenotypes in HFD-Fed Mice

**Figure 7:**
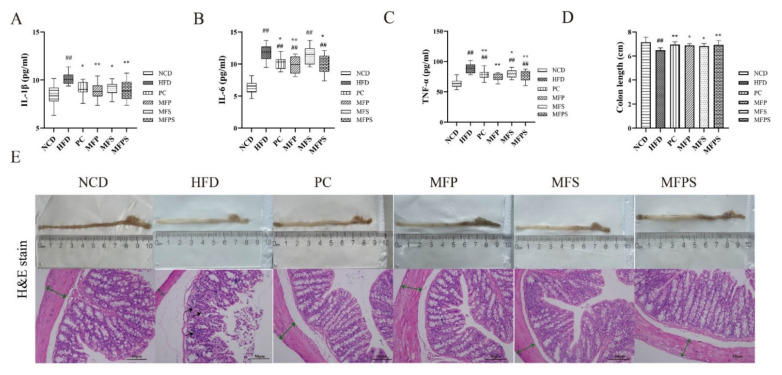
Effects of MFP, MFS and MFPS on inflammatory mediators and colonic lesion phenotypes in HFD-fed mice. Serum levels of pro-inflammatory cytokines by ELISA, including interleukin-1β (IL-1β) (**A**), interleukin-6 (IL-6) (**B**), tumor necrosis factor-α (TNF-α) (**C**). (**D**) The renal index (%). (**E**)The colon tissues and H&E-stained photomicrograph of colon tissue (Original magnification × 200). Black arrows indicate inflammatory cell infiltration, green arrows indicate colonic muscle layer. Data were expressed as the mean ± standard deviation (*n* = 12). *^##^ p* < 0.01 vs. NCD group; * p < 0.05, ** p < 0.01 vs. HFD group.

Diseases associated with MetS, such as IR, NAFLD, and kidney injury, are strongly related to inflammatory cytokines [30]. Levels of IL-1β, IL-6 and TNF-α were notably decreased by treatments with simvastatin, MFP, MFS, MFPS, in comparison with their levels in the HFD group (*p* < 0.05, *p* < 0.01, *p* < 0.05 and *p* < 0.01 for IL-1β; *p* < 0.05, *p* < 0.01, *p* > 0.05 and *p* < 0.05 for IL-6; *p* < 0.01, *p* < 0.01, *p* < 0.05 and *p* < 0.01 for TNF-α) (Figure 7A–C).

As shown in Figure 7D,E, colon length in the HFD group was obviously shorter than that in the NCD group, while the shortening was markedly inhibited by treatments with simvastatin, MFP, MFS and MFPS (*p* < 0.01, *p* < 0.05, *p* < 0.05 and *p* < 0.01). The H&E-stained proximal colons showed that HFD induced impairment of the colonic tissue, including thinned colonic muscle layer, absence of goblet cells, incomplete crypt structure, and inflammatory cell infiltration (Figure 7E). Simvastatin, MFP, MFS and MFPS treatments attenuated the changes in mucosal structure and thickness of the muscle layer. It is suggested that simvastatin, MFP, MFS and MFPS alleviate the colonic lesion phenotypes induced by HFD feeding.

### 3.8. Improvement of Gut Microbiota Dysbiosis in HFD-Fed Mice after MFP, MFS and MFPS Treatments

Recently, increasing evidence has indicated that intestinal dysbiosis is a critical factor in MetS-related diseases such as obesity, NAFLD, and diabetes [31]. In this study, 16S rDNA gene sequencing was performed on feces to explore the potential participation of MFP, MFS and MFPS in mediating the composition of gut microbiota. In total, 1,335,287 valid tags and 9050 OTUs were obtained from 24 fecal samples after quality control (Table S1). Both rank–abundance distribution curves and species accumulation curves flattened out with increasing numbers of sampled sequences; they indicated that the amount of sequencing was sufficient to cover most of the microbial species (Figure S1A,B).

**Figure 8:**
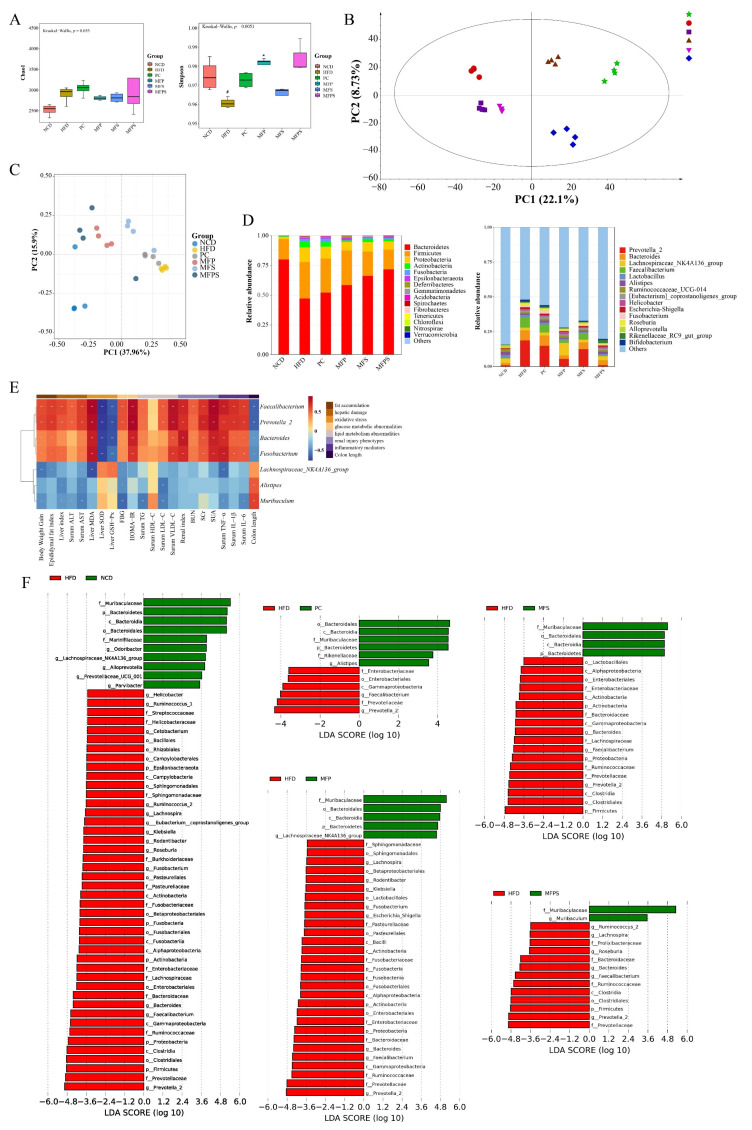
The improvement of gut microbiota dysbiosis in HFD-fed mice after MFP, MFS and MFPS treatments (*n* = 4). (**A**) Boxplots for Chao 1 index and Simpson index. (**B**) OPLS-DA plot. (**C**) PCoA plot. (**D**) Relative abundance of the main phyla and genera of the intestinal microbiota in different groups. (**E**) Spearman’s correlation analysis between gut microbiota genera and pharmacodynamic parameters (The color scale represents the Spearman *r* value, with red and blue indicating positive and negative correlations, respectively. *^#^ p* < 0.05, * *p* < 0.05 and ** *p* < 0.01). (**F**) LEfSe (linear discriminant analysis effect size) (LDA score >3.5).

The Chao 1 index is a parameter used for measuring species abundance, and the Simpson index is a parameter used for estimating species diversity [32]. Among groups, there were no remarkable differences in the Chao 1 index, illustrating that HFD feeding had no influence on the species abundance of the intestinal flora (*p* = 0.053, Figure 8A). Accordingly, the intestinal flora diversity was decreased in the HFD group, indicating that the data could be used for further analysis. The Simpson index had obvious differences among groups, and the HFD group was the lowest (*p* = 0.028, Figure 8A). To reveal the effects of MFP, MFS and MFPS on gut microbial composition, beta diversity of the gut microbiota was investigated using orthogonal partial least squares analysis (OPLS-DA) and principal coordinates analysis (PCoA) based on the distance matrix (Bray–Curtis algorithm) (Figure 8B,C). In OPLS-DA score plots, the gut microbiota of the MFP and MFPS groups was closer to that of the NCD group, whereas the plot of the HFD group visibly shifted along the negative orientation of the first principal component (PC1). HFD-induced gut microbiota dysbiosis was modulated by MFP, MFS and MFPS, and MFP and MFPS groups drove gut microbiota composition similar to that of the NCD group.

To elucidate the composition of gut microbiota, species accumulation histograms of OTUs were analyzed at phylum and genus levels. Bacteroidetes was the most dominant phylum among the sample intestinal bacteria, followed by Firmicutes (Figure 8D). At phylum level, compared with the NCD group, the relative abundances of Firmicutes, Proteobacteria, Actinobacteria, and ratio of Firmicutes/Bacteroidetes were considerably increased in the HFD group, while Bacteroidetes was obviously decreased. At genus level, *Prevotella_2*, *Faecalibacterium*, and *Bacteroides* were notably enriched in the HFD group. Simvastatin, MFP, MFS and MFPS treatments ameliorated the HFD-induced variations in the bacterial community structure (Figure S1C,D).

To further investigate the effects of MFP, MFS and MFPS on gut microbial composition in HFD-fed mice, the gut microbiota at genus level with significant differences among groups was assessed by linear discriminant analysis effect size (LEfSe) (LDA score >3.5). As shown in the cladograms (Figure 8F), HFD-fed mice were featured with genera *Prevotella 2*, *Bacteroides*, *Faecalibacterium*, and *Fusobacterium,* while those in the PC group were featured with *Alistipes*. Mice in the MFP group were characterized by *Lachnospiraceae_NK4A136_group*. The abundance of *Muribaculum* was enriched in the MFS and MFPS groups. It was revealed that changes in the MFP, MFS and MFPS groups were attributed to the growth of beneficial bacteria (*Muribaculum* and *Lachnospiraceae_NK4A136_group*) and reduction of harmful bacteria (*Prevotella 2*, *Bacteroides*, *Faecalibacterium* and *Fusobacterium*) in the intestinal microbiota.

Spearman’s correlation analysis was performed to assess the correlation between perturbed gut microbiota at genus level and pharmacodynamic parameters. The pharmacodynamic parameters were averaged in each cage of mice. As shown in Figure 8E, most of the changed gut microbiota at genus level were positively or negatively correlated with fat accumulation, hepatic damage, oxidative stress, glucose metabolic abnormalities, lipid metabolism abnormalities, renal injury phenotypes, inflammatory mediators and colon length. This finding emphasized the vital role of intestinal microbiota in participating in MetS.

### 3.9. Changes inf Fecal Metabolites in HFD-Fed Mice after MFP, MFS and MFPS Treatments

**Figure 9:**
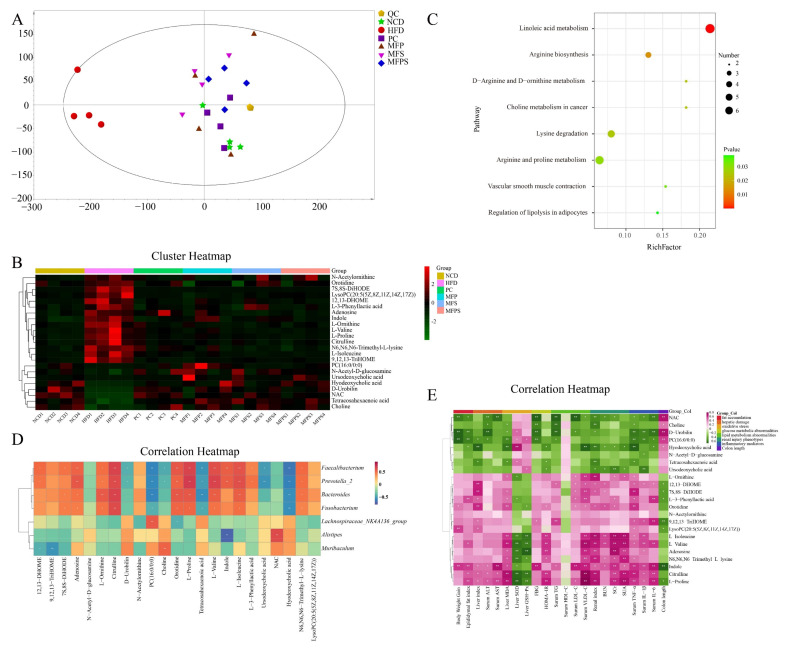
The changes in fecal metabolites in HFD-fed mice after MFP, MFS and MFPS treatments (*n* = 4). (**A**) PCA score plot. (**B**) Cluster heatmap (color bars showing green to red indicate the relative content of metabolites, with red representing high expression and green representing low expression). (**C**) Pathway enrichment analysis (HFD vs. NCD). (**D**) Spearman’s correlation analysis between gut microbiota and different metabolites (color scale represents the Spearman *r* value, with red and blue indicating positive and negative correlations, respectively. * p < 0.05, ** p < 0.01). (**E**) Spearman’s correlation analysis between pharmacodynamic parameters and different metabolites (color scale represents the Spearman *r* value, with pink and green indicating positive and negative correlations, respectively. * p < 0.05, ** p < 0.01).

In order to clarify the potential role of MFP, MFS and MFPS in ameliorating HFD-induced MetS in C57BL/6J mice, untargeted fecal metabolomics analysis was applied to characterize the differences between MFP, MFS, MFPS and HFD groups. Principal component analysis (PCA) and OPLS-DA score plots displayed that obvious separation occurred in fecal samples of different groups (Figure 9A and Figure S2A). It demonstrated that HFD and NCD groups formed two individual clusters. In addition, the clustering of PC, MFP, MFS and MFPS groups was apparently separated from HFD group on the *x*-axis, illustrating that simvastatin, MFP, MFS and MFPS modulated the metabolic disturbances induced by HFD.

With 7-fold cross validation and an interchange test (RPT) of 200-response, permutation plots were generated to investigate the model quality (Figure S2B). Differential metabolites were distinguished depending on Student’s *t*-test (*p* < 0.05) and OPLS-DA model (VIP > 1). The amounts and movements of metabolites were visualized as volcano plots (Figure S1C), and 23 common differential metabolites of HFD vs. NCD, PC vs. HFD, MFP vs. HFD, MFS vs. HFD and MFPS vs. HFD were identified as potential biomarkers (Table S2). Among the aforementioned 23 potential biomarkers (displayed in Table S2 and Figure 9B), 15 potential biomarkers were noticeably up-regulated in the HFD group, and 8 potential biomarkers were prominently down-regulated (*p* < 0.05); moreover, 17, 17, 18, and 20 of them were recovered after PC, MFP, MFS and MFPS treatments (*p* < 0.05), respectively. With the use of MetaboAnalyst 5.0 (https://www.metaboanalyst.ca/, accessed on 16 December 2021), the 23 potential biomarkers were analyzed for metabolic disorder pathways based on KEGG pathway enrichment analysis (Figure 9C). The key altered metabolic pathways of MetS were Linoleic acid metabolism, Arginine biosynthesis, D-Arginine and D-ornithine metabolism, Choline metabolism in cancer, Lysine degradation, Arginine and proline metabolism, Vascular smooth muscle contraction, and Regulation of lipolysis in adipocytes (*p* < 0.05).

Spearman’s correlation analysis was performed to assess the correlations between different metabolites, perturbed gut microbiota at genus level, and pharmacodynamic parameters. As shown in Figure 9D,E, most of the changed gut microbiota at genus levels and pharmacodynamic parameters were positively or negatively correlated with differential metabolites. It was indicated that there were potential correlations between different metabolites and gut microbiota composition and pharmacodynamic parameters.

## 4. Discussion

*Morus alba* L. fruit polyphenols and polysaccharides, as secondary metabolites, are rarely absorbed in small intestine and reach the colon in an almost unchanged form (90–95% of total intake) [33]. In intestinal lumen, the colonic microbiota acts as a major player in decomposing polyphenols into absorbable phenolic metabolites, which exert beneficial effects on microbiota [34]. Polysaccharides, as natural macromolecules isolated from natural sources (e.g., plants, fungi, algae), physically improve intestinal function, regulate the structure of intestinal bacteria, act as substrates for microbial fermentation and protect the immune system [35]. In this study, it was found that MFP, MFS and MFPS improved the gut microbiota composition and fecal metabolite profiles of MetS in HFD-fed mice. In particular, combined application of MFP and MFS exerted stronger effects.

MetS is featured with aberrant glucose metabolism, lipid accumulation and chronic inflammation, especially fat accumulation and NAFLD [36]. In this study, MFP, MFS and MFPS not only ameliorated glucose metabolism disorder (Figure 4A–E) and dyslipidemia in HFD-fed mice (Figure 5A–E), but also alleviated enlargement of epididymal adipocytes (Figure 2D). There is evidence that NAFLD is not only a hepatic manifestation of MetS, but also an important preliminary stage in MetS [37]. Associations between MetS and renal function have been realized; for instance, IR and obesity are risk factors for renal impairment [38]. In this study, pathophysiological alterations were observed in livers and kidneys of HFD-fed mice (Figure 3A–D and Figure 6A–E). After treatments with MFP, MFS and MFPS, hepatic steatosis and vacuolar degeneration in kidney were improved (Figure 3D and Figure 6E). Available evidence supports that oxidative stress is strongly associated with MetS [39]. HFD feeding induced metabolic disturbances by upregulating pro-inflammatory cytokines, which are correlated with NAFLD and renal impairment [40]. In this research, simultaneous analysis of levels of IL-1β, IL-6, TNF-α (Figure 7A–C), MDA, SOD and GSH-Px (Figure 3E–G) provided a comprehensive assessment of oxidative stress levels. TNF-α not only impairs hepatocytes, but also stimulates the production of other inflammatory cytokines (e.g., IL-1β and IL-6), thus causing apoptosis [41]. The ability to scavenge oxygen radicals was assessed by activities of SOD and GSH-Px in liver, while lipid peroxidation was assessed by measuring MDA in liver [42]. In this study, it was indicated that MFP, MFS and MFPS alleviated MetS by improving antioxidant activity.

The host–bacteria relationship is complementary and critical to the maintenance of normal material metabolism, although there is multi-individual diversity in gut microbial composition [43]. Accumulating studies have indicated that gut microbiota plays a crucial role in MetS-related complications such as obesity, NAFLD, IR and T2DM [44]. In this study, the HFD-induced MetS model was successfully established in mice. 16S rDNA sequencing was performed on mice feces to explore whether MFP, MFS and MFPS attenuated MetS via modulating intestinal microbiota. Previous studies have revealed that HFD alters intestinal microbiota composition as well as the gene expression of microorganisms [45]. In this study, consistent with results of many available research works, HFD induced relative abundance of Firmicutes, Proteobacteria, Actinobacteria, Fusobacteria, and the ratio of Firmicutes/Bacteroidetes, and a reduction in Bacteroidetes at phylum level (Figure S1C). More than related, MetS is strongly associated with the ratio of Firmicutes/Bacteroidetes, as gut microbiota reduces Bacteroidetes phylum by obtaining more calories from HFD feeding [46]. *Prevotella_2* enriched in the HFD group is a member of family Prevotellaceae. Previous studies demonstrated that *Prevotella_2* was abundant in the HFD group and positively associated with lifetime cardiovascular disease risk [47]. Studies have found that a high-fat/low-fiber western diet is associated with a predominance of *Bacteroides*-rich gut ecosystem [48]. *Faecalibaculum* belongs to family Erysipelotrichaceae, and previously has been indicated as being abundant in mice fed with “western diet” and strongly related to energy production or fat adiposity [49]. *Faecalibaculum* and *Fusobacterium* are pro-inflammatory bacteria that may impair the gut barrier and are involved in accentuation of hepatic steatosis [41]. *Alistipes* is a member of the phylum Bacteroidetes in family Rickenellaceae. Studies have demonstrated that the abundance of *Alistipes* is negatively correlated with obesity and intestinal redox state [41,50]. Moreover, Alistipes is considered to be a SCFA producer [50], and a reduced abundance of it was observed in patients with NAFLD and liver fibrosis [51]. In contrast, *Alistipes* may be pathogenic in anxiety disorders, chronic fatigue syndrome, and depression, although it exerts a protective role in health phenotype [50]. *Lachnospiraceae_NK4A136_group* belongs to family Lachnospiraceae; it is a butyrate-producing bacterium that has been observed to maintain the integrity of the intestinal barrier in mice [52]. Similarly to this study, the abundance of *Lachnospiraceae_NK4A136_group* was decreased significantly in chronic kidney disease [53] and HFD feeding rats [54]. *Muribaculum* has previously been observed to be negatively correlated with MetS [55]. In this study, simvastatin, MFP, MFS and MFPS treatments modified the overall structure of intestinal microbiota.

Studies have demonstrated a strong association between intestinal microbiota and metabolomics. Intestinal microbiota influences energy expenditure and modulates nutrient metabolism, resulting in altered metabolism of exogenous and endogenous substances [56]. Therefore, the contributions of MFP, MFS and MFPS to fecal metabolites were studied. According to the theory of the gut–liver axis, drugs are fermented by intestinal microbial flora, which leads to changes in metabolites after entering the intestine [57]. Moreover, the intestinal flora ferments undigested proteins and peptides in colon, generating potentially toxic or beneficial components that modulate biological functions [58]. In this study, linoleic acid metabolism, amino acid metabolic pathways and choline metabolism in cancer were markedly altered in the HFD group (Figure 9C). 7S,8S-DiHODE and 9,12,13-TriHOME were down-regulated, while choline and PC (16:0/0:0) were up-regulated after MFP, MFS and MFPS treatments (Table S2). Linoleic acid metabolism is related to obesity and obesity-associated diseases [59]. 7S,8S-DiHODE and 9,12,13-TriHOME are terminal metabolites of the linoleic acid metabolic pathway that play an important role in regulating the immune system and promoting inflammation [60]. Choline, whose level relates to the transport capacity of plasma lipids, helps reduce the accumulation of cholesterol in peripheral tissues [61]. The choline level marks the transport capacity of plasma lipids, and it can reduce the accumulation of cholesterol in peripheral tissues [62]. The decrease in choline may lead to the formation of fatty liver and reduction of the VLDL level [63]. L-arginine, widely present in vivo, is interconvertible with L-proline and participates in synthesis of most proteins [64]. It has been shown that the level of L-arginine is remarkably increased in the presence of renal insufficiency and renal failure, and positively correlated with SCr and BUN [64]. Similarly to previous studies, this study found that the level of L-proline increased significantly in HFD-fed mice (Figure 9C). After treatments with MFP, MFS and MFPS, L-proline returned to normal levels, indicating that MFP, MFS and MFPS can improve renal function by regulating the metabolism of L-arginine and L-proline. Branched chain amino acids (BCAAs), including L-isoleucine, L-leucine and L-valine, are essential amino acids for humans and animals and are involved in various physiological functions [65]. Increased BCAA levels, showing correlations with factors of MetS, have been associated with diabetes, IR, NAFLD and obesity [66]. Recently, it was reported that there may be a compensatory increase in BCAAs in obese individuals under high oxidative stress states [67]. As suggested by Newgard, elevated BCAA levels in serum may result from a decrease in BCAA catabolism associated with obesity. Therefore, in the presence of overnutrition or obesity, the abundance of glucose and lipid substrates makes amino acids less prominent as an energy source, resulting in accumulation [68]. Overall, MFP, MFS and MFPS attenuated the HFD-fed mouse phenotype through reduction of oxidative stress, restoration of intestinal ecological eubiosis, and modulation of the interaction between the intestinal microbiota and host metabolites.

## 5. Conclusions

In summary, polyphenols and polysaccharides derived from *Morus alba* L. fruit attenuated MetS in HFD-fed mice, especially in combination. The potential mechanisms for MFP, MFS and MFPS to treat MetS were reshaping the structure of gut microbiota and restoration of fecal metabolite profiles. Meanwhile, the potential biomarkers and differential gut microbiota in the feces were screened out. Moreover, this is the first study to show that *Morus alba* L. fruit-induced modifications of gut microbiota and fecal metabolite profiles play an important role against MetS. The findings suggest that *Morus alba* L. fruit is a nutritional and functional food for the prevention of MetS, and intestinal flora is the potential drug target.

## Data Availability

The data presented in this study are available on request from the corresponding author.

## Supplementary Materials

The following supporting information can be downloaded at: https://www.mdpi.com/article/10.3390/foods11121818/s1, Figure S1: The improvement of gut microbiota dysbiosis in HFD-fed mice after MFP, MFS and MFPS treatments (*n* = 4), Figure S2: The changes in fecal metabolites in HFD-fed mice after MFP, MFS and MFPS treatments (*n* = 4). (A) OPLS-DA score plots. (B) Permutation plots. (C) Volcano plots, Table S1: Statistics on reads/OTUs for 16S-rDNA sequencing (*n* = 4), Table S2. The 23 common differential metabolites (*n* = 4).

## Abbreviations

MetS, metabolic syndrome; HFD, high-fat diet; NCD, normal chow diet; PC, positive control group; MFP, *Morus alba* L. fruit polyphenols; MFS, *Morus alba* L. fruit polysaccharides; MFPS, *Morus alba* L. fruit polyphenols plus polysaccharides; HPLC, high performance liquid chromatography; BW, body weight; OGTT, oral glucose tolerance test; AUC, area under the curve; HOMA-IR, homeostasis model assessment of insulin resistance; AST, aspartate aminotransferase; ALT, alanine amino transferase; ELISA, enzyme linked immunosorbent assay; MDA, malondialdehyde; SOD, superoxide dismutase; GSH-Px, glutathione peroxidase; H&E, hematoxylin and eosin; OCT, optimal cutting temperature; TC, total cholesterol; TG, triglyceride; LDL-C, low density lipoprotein-cholesterol; HDL-C, high density lipoprotein-cholesterol; NAFLD, nonalcoholic fatty liver disease; SCr, serum creatinine; SUA, serum uric acid; BUN, blood urea nitrogen; IL-1β, interleukin-1β; IL-6, interleukin-6; TNF-α, tumor necrosis factor-α; QCs, quality control samples; OTUs, operational taxonomic units; OPLS-DA, orthogonal partial least squares analysis; PCoA, principal coordinates analysis; LEfSe, Linear discriminant analysis Effect Size; LDA, linear discrimination analysis; PCA, principal component analysis; VIP, Variable important in projection.
