# Supplementary material for: Polyphenols and Polysaccharides from Morus alba L. Fruit Attenuate High-Fat Diet-Induced Metabolic Syndrome Modifying the Gut Microbiota and Metabolite Profile
Source: Foods. 2022 Jun 20;11(12):1818. doi: 10.3390/foods11121818 (PMC9223293; doi:10.3390/foods11121818)
Supplement: Supplementary file 1 [file foods-11-01818-s001.zip › foods-1738743-supplementary.pdf]

foods

Supplementary material

Integrated gut microbiota and untargeted fecal metabolomics reveal beneficial effects of polyphenols and polysaccharides from Mori Fructus (*Morus alba* L.) on HFD-induced metabolic syndrome in C57BL/6J mice

Meixia Wan [a, b], Qing Li [a], Qianya Lei [a], Dan Zhou [a], Shu Wang [a, *]

a: West China Sch Pharm, Sichuan University, No.17, Section 3, Renmin Nan Rd, Chengdu, 610041, Sichuan, Peoples R China

b: Qibo college of medicine, Longdong University, No.45, Lanzhou Rd, Qingyang, 745000, Gansu, Peoples R China

*Corresponding author: Shu Wang, West China Sch Pharm, Sichuan University, No.17, Section 3, Renmin Nan Rd, Chengdu, 610041, Sichuan, Peoples R China, TEL: (86) 028-85503950, E-mail: suwang571@126.com.

Table S1. Statistics on reads/OTUs for 16S-rDNA sequencing (n = 4)

Sample	clean_tags	valid_tags	valid mean Length	OTU_counts	Total_OTUs
NCD1	67177	54482	417.88	2351	9050
NCD2	68985	58669	417.09	2065	9050
NCD3	62971	55051	416.58	2193	9050
NCD4	67591	57226	417.93	2181	9050
HFD1	64450	55558	405.43	2917	9050
HFD2	69896	59889	408.1	2987	9050
HFD3	62745	52514	410.63	2763	9050
HFD4	60007	50370	400.57	2469	9050
PC1	66245	56643	408.65	3084	9050
PC2	66919	56989	412.31	2569	9050
PC3	59201	48767	411.72	2699	9050
PC4	67422	56757	408.87	2944	9050
MFP1	66119	58783	415.65	2443	9050
MFP2	67568	60820	413.26	2428	9050
MFP3	65447	58552	413.75	2490	9050
MFP4	67376	59277	415.27	2472	9050
MFS1	65807	58489	415.63	2501	9050
MFS2	66249	58489	415.35	2636	9050
MFS3	62763	56209	415.86	2469	9050
MFS4	70213	60754	410.97	2887	9050
MFPS1	64023	56974	416.61	2417	9050
MFPS2	38604	29773	404.16	3318	9050
MFPS3	65975	57040	417.31	2639	9050
MFPS4	63803	57212	418.87	2035	9050

OTUs, operational taxonomic units

Table S2. The 23 common differential metabolites (n = 4)

**m/z**	**RT (min)**	**Ion mode**	**Metabolites**	**Formula**	**VIP**	**HFD vs NCD**	**PC vs HFD**	**MFP vs HFD**	**MFS vs HFD**	**MFPS vs HFD**
542.3256	6.13	pos	LysoPC(20:5(5Z,8Z,11Z,14Z,17Z)	C28H48NO7P	1.03	↑ #	_	_	↓ *	_
189.1597	0.63	pos	N6,N6,N6-Trimethyl-L-lysine	C9H20N2O2	1.08	↑ ##	_	_	↓ **	↓ **
118.0653	4.20	pos	Indole	C8H7N	1.61	↑ ##	↓ **	_	↓ **	↓ **
133.0972	0.61	pos	L-Ornithine	C5H12N2O2	1.61	↑ ##	↓ *	↓ *	↓ **	↓ *
335.2196	9.58	pos	7S,8S-DiHODE	C18H32O4	1.80	↑ ##	↓ **	↓ **	↓ **	↓ **
283.1442	4.94	pos	NAC	C17H18N2O2	1.84	↓ ##	↑ **	↑ **	↑ **	↑ **
175.1077	0.72	pos	N-Acetylornithine	C7H14N2O3	2.14	↑ ##	↓ *	↓ *	_	_

268.1038	1.18	pos	Adenosine	C10H13N5O4	4.04	↑ ##	_	_	↓ **	↓ **
589.3016	7.59	pos	D-Urobilin	C33H40N4O6	4.95	↓ ##	↑ **	↑ *	↑ **	↑ **
118.0865	1.08	pos	L-Valine	C5H11NO2	6.45	↑ #	↓ *	↓ *	↓ *	↓ *
104.1074	0.66	pos	Choline	C5H13NO	7.02	↓ ##	_	↑ *	↑ *	↑ **
176.1029	0.67	pos	Citrulline	C6H13N3O3	8.05	↑ #	↓ *	↓ *	↓ *	↓ *
353.2298	9.12	pos	9,12,13-TriHOME	C18H34O5	8.26	↑ ##	↓ **	↓ **	↓ **	↓ **
116.0708	0.72	pos	L-Proline	C5H9NO2	10.62	↑ #	↓ *	↓ *	↓ *	↓ *
357.2784	10.83	pos	Tetracosahexaenoic acid	C24H36O2	17.89	↓ ##	↑ **	↑ **	↑ **	↑ **
132.1020	1.36	pos	L-Isoleucine	C6H13NO2	28.11	↑ ##	↓ **	↓ **	↓ **	↓ **
269.0416	4.67	neg	Orotidine	C10H12N2O8	1.58	↑ #	↓ *	↓ *	_	↓ *
66.0881	0.73	neg	N-Acetyl-D-glucosamine	C8H15NO6	2.43	↓ #	_	_	_	↑ *
313.2386	10.41	neg	12,13-DHOME	C18H34O4	4.56	↑ ##	↓ **	↓ *	↓ *	↓ **
540.3310	11.22	neg	PC (16:0/0:0)	C24H50NO7P	5.05	↓ #	_	↑ *	↑ *	↑ *
437.2910	9.71	neg	Ursodeoxycholic acid	C24H40O4	5.15	↓ ##	↑ **	↑ *	_	_
165.0547	5.50	neg	L-3-Phenyllactic acid	C9H10O3	5.28	↑ #	↓ *	↓ *	_	↓ *
783.5786	9.85	neg	Hyodeoxycholic acid	C24H40O4	7.31	↓ #	↑ **	_	↑ *	↑ **

RT, retention time; VIP, variable importance of projection; Data were expressed as the mean ± standard deviation (n = 4). $^{\#}P < 0.05$, $^{\#\#}P < 0.01$ vs. NCD group; $^{*}P < 0.05$, $^{**}P < 0.01$ vs. HFD group. ' ↑ ' and ' ↓ ' indicate metabolites with an upward or downward trend. # and ## represent compared with NCD group, $^{\#}P < 0.05$, $^{\#\#}P < 0.01$, respectively. * and ** represent compared with HFD group, $^{*}P < 0.05$, $^{**}P < 0.01$, respectively.

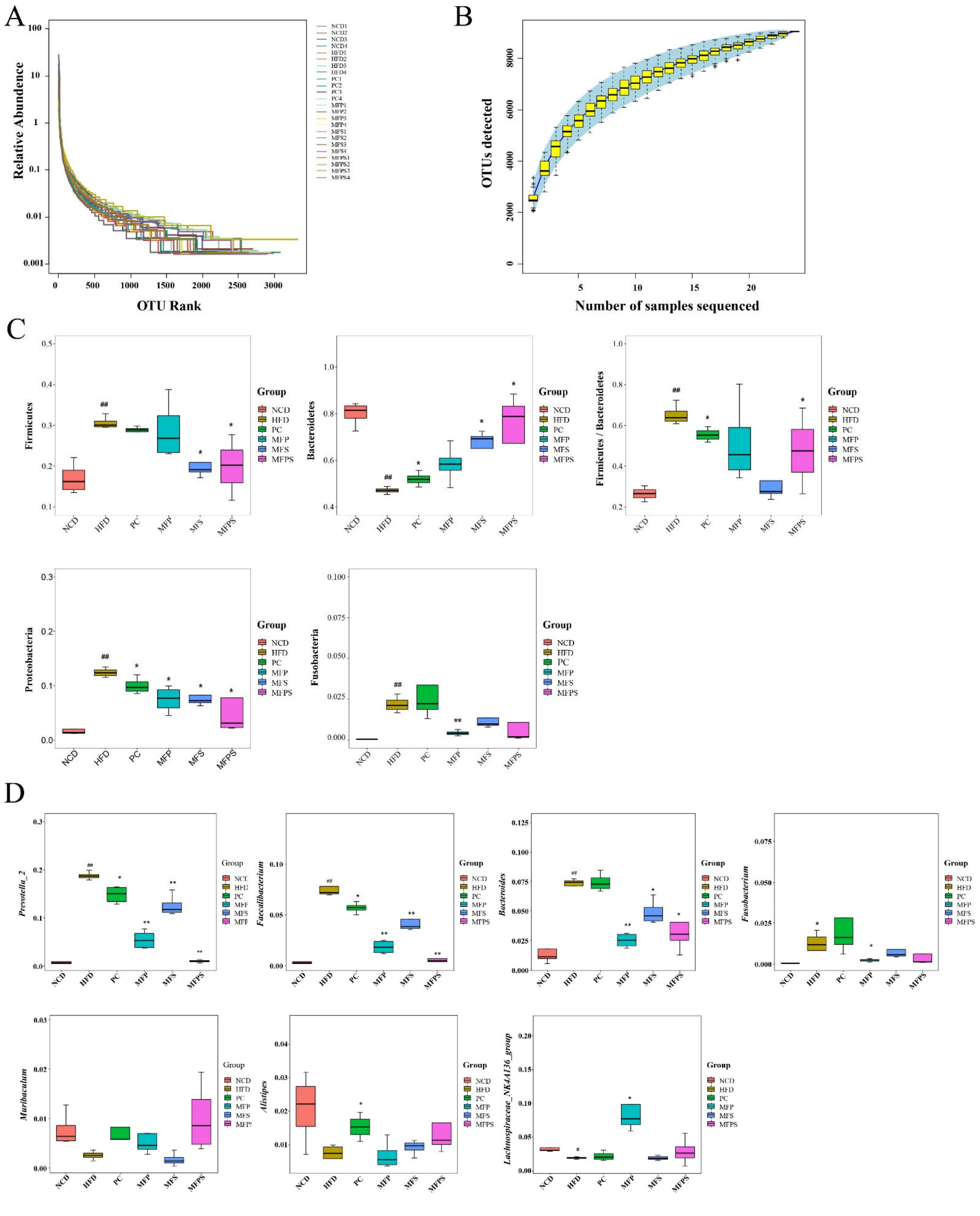

Figure. S1. The improvement of gut microbiota dysbiosis in HFD-induced mice after MFP, MFS and MFPS treatments (n = 4). (A) Rank-abundance distribution curve. (B) Species accumulation

curves. (C and D) Taxonomic analysis was performed to analyze the effects of MFP, MFS and MFPS on composition of gut microbiota at phylum (C) and genus. $^{\#}P < 0.05$, $^{\#\#}P < 0.01$ vs. NCD group; $^{*}P < 0.05$, $^{**}P < 0.01$ vs. HFD group.

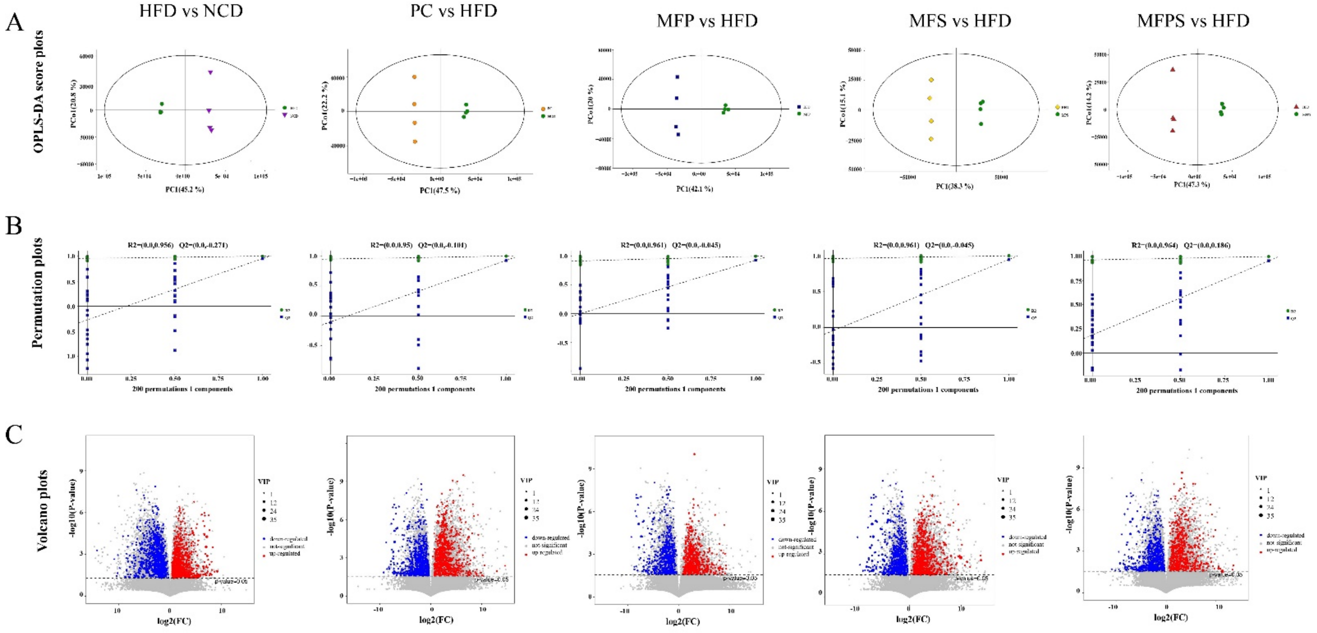

Figure. S2. The changes of fecal metabolites in HFD-induced mice after MFP, MFS and MFPS treatments (n = 4). (A) OPLS-DA score plots. (B) Permutation plots. (C) Volcano plots.
